# Analysis of Teaching Tactics Characteristics of Track and Field Sports Training in Colleges and Universities Based on Deep Neural Network

**DOI:** 10.1155/2022/1932596

**Published:** 2022-08-21

**Authors:** Wei Wang

**Affiliations:** Sports Department, Wuhu Institute of Technology, Anhui Wuhu 241003, China

## Abstract

In the analysis of the teaching tactical characteristics of track and field sports training in colleges and universities, the teaching tactical characteristics are not quantified, which leads to the low key degree of determining the influencing factor indicators in colleges and universities, and the error in the evaluation of the teaching tactical characteristics of track and field sports training is large. Therefore, this paper designs a method to analyze the tactical characteristics of college track and field sports training teaching based on deep neural network. Firstly, by analyzing the current situation of track and field sports training teaching in colleges and universities, it determines the areas that need to be improved in teaching. Then, by determining the factors of teaching environment, the core competitiveness of track and field teams, and the teaching ability of track and field coaches, these factors are determined as the key characteristics, the data basis is analyzed, and the unified data quantitative processing is carried out to determine the key factor indexes affecting the analysis of tactical characteristics. Finally, the deep neural network is introduced to construct the evaluation model of the tactical characteristics of college track and field sports training teaching, and the characteristic analysis results are further modified with the help of cascade noise reduction self-encoder to complete the analysis of the tactical characteristics of college track and field sports training teaching. The experimental results show that the proposed method can effectively analyze the teaching tactical characteristics of track and field sports training in colleges and universities and improve the performance of the evaluation of the teaching tactical characteristics of track and field sports training.

## 1. Introduction

College track and field training is an important part of school physical education. Track and field is one of the key items of school physical education teaching and after-school training. It has the advantage of talent concentration and wide popularity [1, 2]. Track and field training is the basic training stage of track and field. It is the key stage to develop sports quality, learn and master track and field technology, and improve track and field ability. Paying attention to and strengthening track and field training is an important link to improve the overall training level of track and field [3]. Track and field sports play a positive role in realizing the knowledge, technology, and skills of sports in college physical education and improving students' physical and psychological quality and social adaptability. However, in the process of deepening the reform of physical education teaching in colleges and universities, it is found that the number of students participating in track and field is becoming less and less, and the students' understanding of the value of participating in track and field is becoming less and less. The school also makes track and field teaching more and more marginalized because of the students' weak awareness of track and field teaching [4]. At the same time, track and field teaching pursues the integrity and systematization of competitive events too much and only pays attention to the teaching of technical actions in running, jumping, throwing, and other events, and the teaching means are single. It can not develop students' relevant physical quality around a certain event, so students' subjectivity and creativity are difficult to play. Only by making the track and field teaching concept clear and the goal clear and paying attention to a series of problems such as teaching content, teaching methods, and textbook selection, can the track and field teaching reform in colleges and universities not be in a chaotic and fuzzy state and can a clear main line be provided for the reform and development of track and field teaching in colleges and universities [5, 6].

How to cultivate track and field sports talents who can shoulder the historical mission and have practical ability and innovation ability, constantly keep pace with the times, improve track and field sports training tactics, implement effective training tactics, and improve the quality of track and field training has become the top priority to be solved by China's colleges and universities of physical education [7]. Therefore, relevant researchers have made a lot of analysis on the tactical characteristics of track and field training in colleges and universities. Literature [8] designed a simulation training method and studied its application in track and field training tactics. In this technology, in order to ensure that athletes can play a better level in the competition, coaches should not only strengthen the training of their physical quality, but also let them fully master and use sports technology, which is a vital way and means in training. Once the psychological and special situation handling skills training is ignored, it will affect the normal competition of athletes and even lead to the loss of the competition. Therefore, this paper mainly analyzes the main advantages and characteristics of track and field simulation training and puts forward the specific application of simulation training method in track and field situation. It aims to promote athletes to have a good psychological and physical state in the process of competition, so as to give full play to their best potential and level and finally win the ideal results. This study pointed out the psychological characteristics and sports characteristics of training technology but did not effectively quantify the detailed tactical characteristics and indicators. Literature [9] analyzes the influencing factors of physical fitness training in track and field sprint events. Take this as the research basis to improve the quality of training tactics. This paper points out that, with the rapid development of track and field sprints in China, the requirements for sprinters' competition skills and tactical level are becoming higher and higher. How to improve the quality of track and field sprinters' training plan and how to quickly enhance the physical quality of track and field sprinters have become the most concerned topic in the field of track and field sprinters' training. Physical training is an important basis for all skill training. In order to make sprinters give full play to their potential and win the sprint competition, physical training is the most indispensable and important factor. This paper will deeply analyze and explore the influencing factors and related contents of physical fitness training in track and field sprint. However, this article is highly theoretical and needs to further expand the main technical characteristics of the research.

In view of the shortcomings in the above analysis of the teaching tactical characteristics of track and field sports training, this paper designs an analysis method of the teaching tactical characteristics of track and field sports training in colleges and universities based on the deep neural network algorithm to complete the analysis of the teaching tactical characteristics of track and field sports training in colleges and universities. The main technical route of this paper is as follows:

  Step 1: by analyzing the current situation of track and field sports training teaching in colleges and universities, determine what needs to be improved in teaching.
  Step 2: by determining the factors of teaching environment, the core competitiveness of track and field teams, and the teaching ability of track and field coaches, these factors are determined as the key characteristics, the data basis is analyzed, and the unified data quantitative processing is carried out to determine the key factor indicators affecting the analysis of tactical characteristics.
  Step 3: introduce the deep neural network to construct the evaluation model of the tactical characteristics of college track and field sports training teaching, and further modify the characteristic analysis results with the help of the cascade noise reduction self-encoder to complete the analysis of the tactical characteristics of college track and field sports training teaching.
  Step 4: conduct experimental analysis. Taking a university as an example, the effectiveness of the proposed analysis method is verified.

## 2. Current Situation of Track and Field Physical Training Teaching in Colleges and Universities and the Determination of Influencing Factors and Indicators of Tactical Characteristics

### 2.1. Analysis on the Current Situation of Track and Field Physical Training Teaching in Colleges and Universities

#### 2.1.1. The Meaning of Track and Field Training Is Vague

The goal of track and field teaching in colleges and universities is to enhance students' physical qualities, help students form good physical exercise habits, and establish correct health awareness. However, in the actual teaching and training, many teachers pay too much attention to the cultivation of students' track and field skills, and take the learning results of track and field skills as the standard to measure students, but they lack accurate positioning for the cultivation of students' fitness consciousness and exercise consciousness. Due to the overemphasis on the integrity and technicality of track and field events in teaching activities and the neglect of students' acceptance ability, students' interest in track and field training is not high [10].

#### 2.1.2. The School Pays Less Attention to the Teaching Value of Sports Track and Field

As a special field, the school field is a space with its own logic and inevitability composed of various objective relations including organization and system. Its interior follows its own unique logic, operation rules, and development track and has a restrictive effect on the survival and development of school leaders and track and field teachers in the field. School field is a realistic factor that affects the value orientation of track and field teaching in colleges and universities. It is mainly reflected in the fact that the school field has more and more successfully made people learn fast, learn more, and learn well, but it has caused the forgetting of the meaning of “learning,” which is not used by people, makes people subordinate to “learning,” is trapped by “learning,” and suppresses the perfection and harmonious generation of people. Track and field classroom teaching is based on rational knowledge. The real life, quality of life, and life value of teachers and students are usually ignored, forgetting the humanistic care for teachers and students and the improvement of human nature [11].

#### 2.1.3. The Organization Form of Track and Field Teaching Is Single

The organizational form of track and field teaching in colleges and universities is relatively single. Teachers often do demonstration actions, then explain step by step, and learn to imitate and practice repeatedly. In this form of teaching organization, teachers are the main body of classroom teaching, and students can only passively accept teachers' knowledge indoctrination, so students' learning enthusiasm is greatly affected. In addition, many teachers only pay attention to how much they have taught but ignore how much students have learned. As long as their teaching tasks are completed, they think they have achieved the purpose of teaching.

#### 2.1.4. The Influence of the Inertia of Power Field on the Occupation of Track and Field Teaching Skills

As an objective social existence, the behavior and thought of educators and other educational participants are affected by the right field, and anyone's survival and development are in a certain right field. Zimmer, a famous sociologist, pointed out that, in any situation of social interaction, people may have the difference between superior position and inferior position. He called this form of social relations with the advantages and disadvantages of position and position as “domination”; that is, the dominant person has the ability and opportunity to influence, decide, and control the inferior person. Looking at the track and field teaching environment of physical education departments in colleges and universities from the field of power, as long as we understand the power of the “test baton,” we can understand why the first place of skills is virtual, and only the students' track and field standard score is real [12].

### 2.2. Determination of Influencing Factors and Indexes of Teaching Tactics of Track and Field Sports Training in Colleges and Universities

In order to more effectively highlight the effectiveness of this analysis method, first quantify the data on track and field sports training teaching characteristics in colleges and universities, convert it into certain data, and more intuitively analyze the tactical characteristics of track and field sports training teaching in colleges and universities. In the teaching of track and field sports in colleges and universities, the teaching environment is the key to the tactical characteristics of track and field sports training and teaching in colleges and universities. Therefore, this paper determines the important indicators affecting the track and field teaching environment in colleges and universities and effectively quantifies them [13].

**Figure 1:**
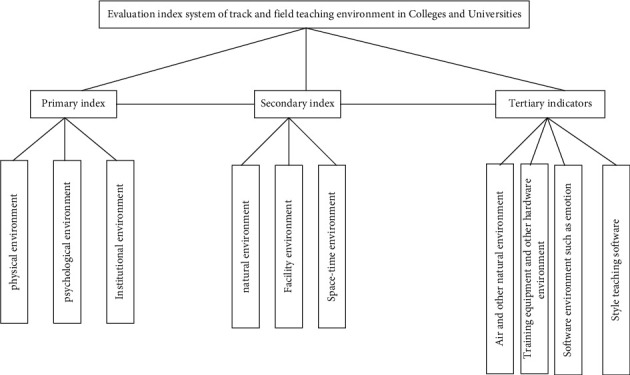
Evaluation index system of track and field teaching environment in colleges and universities.

Taking the element structure system of track and field teaching environment in colleges and universities as the basis, according to the establishment principles of the evaluation system and drawing on previous research experience, this paper summarizes, divides, and compares the important indicators affecting the track and field teaching environment in colleges and universities, forms the conceptual indicators of track and field teaching environment in colleges and universities, and finally constructs the evaluation index system of track and field teaching environment in colleges and universities. The specific contents are shown in Figure 1.

According to the abovementioned evaluation index system of track and field teaching environment in colleges and universities, this teaching strategy is quantitatively analyzed. Because the track and field teaching environment is the main influencing factor, it can not directly transform the data. Therefore, with the help of coefficient of variation, the influencing factors are studied quantitatively. The coefficient of variation is the ratio of the standard deviation of the index to the weighted average. If the coefficient of variation is small, it means that the expert evaluation results are not dispersed or the degree of dispersion is small [14]. Generally, the coefficient should be less than the standard of 0.25. If it is more than or equal to, it means that the index lacks coscheduling. The calculation formula of the influencing factor index is

(1) $$\begin{array}{c} R_{r}=\frac{A_{i}}{v_{i}}. \end{array}$$

Among them, *R*_*r*_ represents the coefficient of variation of track and field teaching environment factors. The smaller the value, the higher the coordination degree of the teaching environment on tactics. *A*_*i*_ represents the standard deviation of teaching environment influencing factors, and *v*_*i*_ represents the arithmetic mean of environmental factors.

In order to minimize the influence of the teaching environment of university track and field on the teaching tactics, the coordination coefficient is introduced to balance it. The meaning of the coordination coefficient is whether there is a large difference between the recommendations in the expert group. The accounting of the coordination coefficient can obtain the degree of expert coordination of the index. The coordination coefficient is represented by *W*, and the value is between 0 and 1. Use consistency test; if *P* > 0.05, the evaluation made by experts does not have strong credibility and the result is invalid; if *P* < 0.05, it has certain credibility and can be used. The calculation formula is

(2) $$\begin{array}{c} P=\frac{W}{\sum R_{r}}. \end{array}$$

According to the above calculation, the influence degree of track and field teaching environmental factors in colleges and universities is determined, which is regarded as a key factor affecting the characteristics of sky landing teaching tactics. In addition, it also includes other influencing factors, which also need to be quantified one by one.

**Figure 2:**
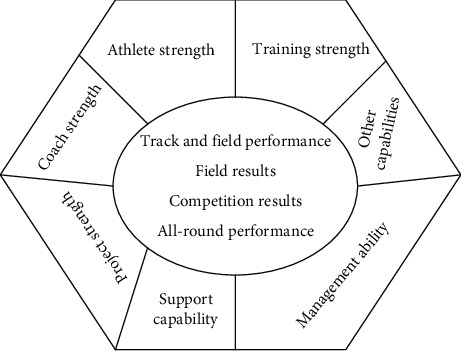
Schematic diagram of influencing factors of core competitiveness of college track and field teams.

Core competitiveness is the integration of various advantageous resources that have been continuously optimized and improved to form their own unique and difficult to imitate form. Its core competitiveness is not only different from its general competitiveness, but also related. The core competitiveness of high-level track and field teams is finally formed in the general competitiveness. It goes beyond the general competitiveness [15], so that it can maintain a stable, leading position and advantage in the field of track and field competition for a long time. The biggest feature of this difference is that it is not easy to be imitated by competitors and can form its own unique competitive advantage. Therefore, to analyze the elements of the core competitiveness of high-level track and field teams, we should extract the most core factors from many “seemingly” general competitiveness elements, because these factors are most likely to play a vital and irreplaceable role in the formation of the core competitiveness of high-level track and field teams in colleges and universities [16]. The schematic diagram of competitive core strength of college track and field teams is shown in Figure 2.

By analyzing Figure 2, it can be found that the current model has the following important characteristics: first, integrity. The model reflects the main components of the core competitiveness of excellent high-level track and field teams, and the content system is relatively complete. The second is scientific. The construction basis of this model is in line with the purpose and law of high-level track and field teams, the basic theory of core competitiveness construction, and the reality of running high-level track and field teams. It is highly scientific. The third is hierarchy, which is divided into three interrelated levels according to the relationship with competitive performance, reflecting the hierarchical principle of system theory, with rigorous logic and clear hierarchy. The fourth is the development and variability. This model expresses the meaning of the continuous operation of various elements of core competitiveness around the “expression of competitive achievements” and suggests that the composition of core competitiveness will change with time and team running conditions. Only when the joint force of these elements can bring competitive advantages to the sports team, can the status of core competitiveness of various elements be stabilized; otherwise it may fall into the ranks of general competitiveness. Therefore, it has development and variability. In the quantification of the influencing factors, the importance of an aspect of the previous level is compared according to the factors in the same level, and the pairwise comparison judgment matrix is completed [17]. When the above layer factors are regarded as standards, the comparative standard *b*_*ij*_ can be adopted to represent the importance of competitiveness influencing factors. The judgment matrix composed is

(3) $$\begin{array}{c} B=\sum_{i=1,j=1}b_{ij}. \end{array}$$

The constructed judgment matrix is tested, and the consistency and randomness of the matrix are tested. The following results are obtained:

(4) $$\begin{array}{c} C=\frac{c_{i}}{\eta_{i}}, \end{array}$$

(5) $$\begin{array}{c} c_{i}=\frac{\lambda \max-k}{k-1}, \end{array}$$

where *η*_*i*_ represents the average random consistency criterion and *c*_*i*_ is a constant.

**Figure 3:**
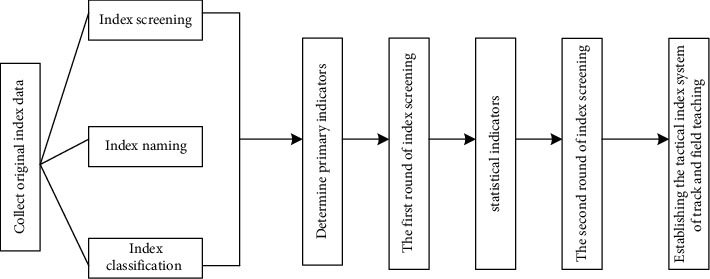
Process of teaching ability index system of track and field coaches.

In the analysis of the tactical characteristics of track and field teaching in colleges and universities, the teaching ability of teachers, that is, coaches in track and field training, is also the key factor affecting the tactical characteristics. When evaluating the teaching ability of track and field coaches, we need to reflect their ability level through multiple indicators. The first step of this study is to classify the collected indicators. The second step is to screen and integrate the indicators with the same connotation and overlapping contents and rename them. On the basis of formulating the primary indicators, the Delphi method (the first round of expert questionnaire) is used to determine the candidate indicators of track and field coaches' teaching ability. Then make statistics of the feedback and further sort out and screen the corresponding indicators to determine the teaching ability index system of track and field coaches. The process is shown in Figure 3.

The contents of the teaching ability indicators of track and field coaches are shown in Table 1.

According to the abovementioned factors affecting the characteristics of track and field teaching tactics in colleges and universities, that is, the factors of teaching environment, the core competitiveness of track and field teams, and the teaching ability of track and field coaches, these factors are determined as the data basis of key characteristic analysis and unified data quantitative research [18]. The set of factors affecting the evaluation objectives is determined as follows:

(6) $$\begin{array}{c} E=\left\{e_{1},e_{2},\ldots ,e_{n}\right\}. \end{array}$$

The comment set for establishing impact assessment objectives is

(7) $$\begin{array}{c} U=\left\{u_{1},u_{2}\ldots u_{n}\right\}. \end{array}$$

Calculate the weight of each evaluation factor and the objective vector of impact assessment is

(8) $$\begin{array}{c} Q=\left\{q_{1},q_{2},\ldots q_{n}\right\}. \end{array}$$

Using analytic hierarchy process to calculate the weight vector: carry out single factor fuzzy evaluation and determine the fuzzy relationship matrix; combined with the single factor evaluation vector of membership degree, complete the construction of multi-index comprehensive evaluation vector, and obtain

(9) $$\begin{array}{c} H_{i}=V\sum_{i=1}^{n}\left(E,U,Q\right). \end{array}$$

According to formula (9), the results of fuzzy comprehensive evaluation are analyzed, and the quantitative influencing factors of track and field teaching tactical characteristics are taken as the key indicators of subsequent characteristic analysis. On this basis, the analysis of track and field teaching tactical characteristics in colleges and universities is improved.

## 3. Design of Evaluation Model of Teaching Tactics Characteristics of Track and Field Physical Training in Colleges and Universities Based on Deep Neural Network

### 3.1. Determination of Teaching Tactics Characteristics of Track and Field Physical Training in Colleges and Universities

Among the tactical characteristics of track and field teaching in colleges and universities, the characteristic of speed change is the external form of tactical application. The smooth implementation of tactics is reflected by athletes through the whole process of speed distribution and speed saving. Therefore, it can be said that the characteristics of speed change are the basis of tactical application. The main features are the following teaching tactics.

#### 3.1.1. Leading Tactics

According to the different positions of athletes in the competition, the tactics can be divided into leading walking tactics and following walking tactics. The leading walking strategy refers to the strategy that athletes start walking at a faster speed at the beginning of the game, strike first, try their best to get rid of the opponent's follow, open the distance with the opponent, and lead all the way through the whole process [19]. The advantage of this tactic is that it can give full play to the physical, tactical, and psychological advantages of athletes without interference from opponents. It is suitable for athletes with good speed endurance, strong ability to walk at high speed for a long time, and special strength. The disadvantage is that the tactical speed changes greatly, the physical energy consumption is much, and the use is too risky. Therefore, it is not recommended to use it by ordinary athletes. If it is used blindly, it will often lead to the second half or final rush due to unreasonable physical distribution.

In the stabbing stage, the physical strength is overdrawn, resulting in the situation of being surpassed by the opponent.

#### 3.1.2. Follow Tactics

Following tactics refers to the tactics that athletes use faster speed to quickly seize a favorable position at the beginning of the competition, closely follow an athlete who is roughly consistent with their own rhythm and pace, and in the final sprint stage, attack later and surpass the follower or even the leader with the advantage of their own sprint ability. The advantage of using this tactic is that athletes only need to concentrate on following the followers, which not only reduces their psychological pressure, but also causes greater psychological pressure to the followers. In addition, from the perspective of hydrodynamics, compared with the follower, the follower resists certain air resistance because the follower leads in front, which makes the follower walk more labor-saving and less physical consumption. It is suitable for athletes with good speed and strong sprint ability, as well as competition in bad weather such as wind and rain [20]: its disadvantage is that the follower's speed rhythm is easily disturbed by the follower. It is easy to put the follower in a passive position on the competition field.

#### 3.1.3. Uniform Velocity Tactics

According to the speed changes of athletes in the competition, tactics can be divided into constant speed tactics and variable speed tactics. Uniform speed tactics refers to the tactics that athletes start and walk at a relatively uniform speed from the beginning of the game and maintain this speed to complete the whole process. The advantage of this tactic is that athletes can determine the speed and rhythm according to their own competitive ability, and the physical consumption is small. It is suitable for athletes who create their best sports performance.

#### 3.1.4. Variable Speed Tactics

Variable speed tactics refers to the tactics that athletes occupy a favorable position at a relatively fast speed at the beginning of the game, constantly adjust their physical fitness through speed control and rhythm changes, interfere with the opponent as much as possible, get rid of the opponent, and win the game. The advantage of this tactic is that it can not only disrupt the opponent's speed change rhythm and break the functional balance of its internal organs and systems, but also cause greater psychological pressure on the opponent. It is suitable for athletes with high speed and endurance. Its disadvantage is that it consumes a lot of physical energy for athletes. If you cannot reasonably distribute your physical strength, it is easy to cause your own physical weakness.

### 3.2. Evaluation Model of Teaching Tactics Characteristics of Track and Field Sports Training in Colleges and Universities

According to the above extracted quantitative indicators of the influencing factors of the teaching tactical characteristics of college track and field sports training, in order to realize the analysis of the teaching tactical characteristics of college track and field sports training, this paper introduces the deep neural network algorithm [21] to construct the evaluation model of the teaching tactical characteristics of college track and field sports training and takes the quantitative indicators of the influencing factors of the teaching tactical characteristics of college track and field sports training as the input data of the neural network, in order to complete the analysis of the teaching tactical characteristics of track and field sports training in colleges and universities.

#### 3.2.1. Theoretical Basis of Deep Neural Network Algorithm

**Figure 4:**
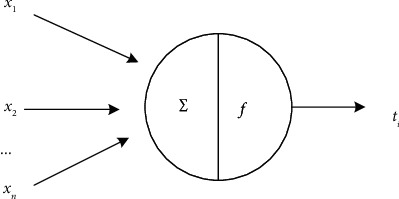
Structure diagram of basic neural network.

Deep neural network is an important branch of machine learning. Deep learning method has the ability to extract abstract features. Compared with machine learning, it does not need to extract features manually but automatically extracts basic features from samples. Then, some more advanced features are extracted layer by layer, such as lines, local structure, and so on. Finally, effective fitting results are obtained by updating model parameters similar to machine learning. The basic neural network structure is shown in Figure 4.

In Figure 4, the circle in the graph represents a neuron, *x*_*i*_ represents the *i* th input signal, *z*_*ij*_ represents the weight of the signal effect on the neuron *j* to simulate the strength of the synapses, *μ*_*ij*_ represents a threshold, and *y*(*x*) represents the activation function after the neuron reaches the threshold, requiring that its nonlinearity is differentiable. The last current layer *i* th output signal *t*_*i*_ may be expressed as follows:

(10) $$\begin{array}{c} t_{i}=y\left(\sum_{i=1}^{n}z_{ij}x_{i}+\mu_{ij}\right). \end{array}$$

The neural nodes in each layer of the traditional network model are connected with each other. As the learning task becomes more and more complex, the performance of hardware equipment shows a certain bottleneck. The amount of network parameters is limited, the neuron layer cannot be further deepened and widened, and the learning ability of the model is limited. In addition, in the field of computer vision, the fully connected network can not use the location information between pixels, which also limits the learning ability of the network to a certain extent. The emergence of deep neural network solves the above problems well [22].

#### 3.2.2. Evaluation on Teaching Tactics of Track and Field Sports Training

With its excellent characteristic learning ability, deep neural network has become one of the most far-reaching achievements in the field of deep learning. For one of the neural network layers, the output characteristic formula is

(11) $$\begin{array}{c} x_{j}^{l}=f\left(\sum_{i=1}^{n}x_{j}^{l-1}\times w_{ij}+\beta_{ij}\right), \end{array}$$

where *w*_*ij*_ represents the ensemble of neurons in the input layer, *x*_*j*_^*l*^ represents the output of the *i* neuron, also the input in layer 1, and *β*_*ij*_ is the bias of the *j* input in layer 1.

Taking the tactical characteristic index data of track and field teaching in colleges and universities as the basic data of initial training in deep neural network, an appropriate loss function is determined to measure the error between the output of deep neural network and the original data [23, 24], and the following results are obtained:

(12) $$\begin{array}{c} W=\sum \frac{1}{2}{\left\|s_{i}-y\right\|}_{2}^{2}, \end{array}$$

where *s*_*i*_ represents the network output value, and *y* represents the sample label value. The network output with the sample labels was fitted by the algorithm to minimize the error.

The multilayer perceptron in the deep neural network further defines the error of the value of track and field teaching tactical characteristics output by the output layer as

(13) $$\begin{array}{c} \varphi_{i}=\frac{\partial w}{\partial s_{i}}Ηf^{\prime }\left(s_{i}\right), \end{array}$$

where *ϕ*_*i*_ represents the inactive output; *H* is the product of Hadama (Hadamard), and *f*′ represents the derivative of the activation function, producing different types depending on the selected activation function.

Then, the gradient descent method [25] is used to update the result value of the tactical characteristics of track and field teaching in colleges and universities, and the following results are obtained:

(14) $$\begin{array}{c} \Delta \varphi_{i}=-\chi \frac{\partial w}{\partial s_{i}}, \end{array}$$

where *χ* represents the learning rate. Through this method, the deep neural network parameters are iteratively updated to solve the target threshold, that is, the key value to determine the tactical characteristics of track and field teaching.

**Figure 5:**
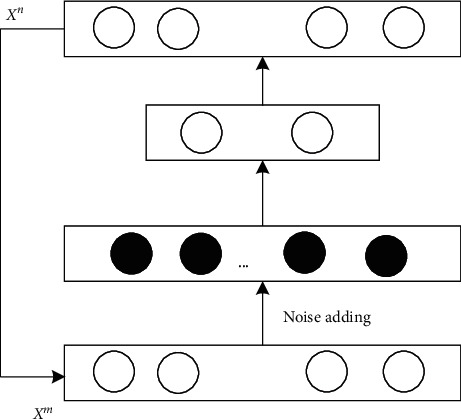
Basic mode of stacked noise reduction self-encoder.

Because the characteristic of track and field teaching tactics is an abstract concept, it can not be output intuitively. Therefore, according to the output results of deep neural network, an unsupervised method is used to extract the key tactical characteristics from a large number of track and field tactical characteristics data. In this paper, the cascade noise reduction self-encoder is introduced to effectively extract the key features of the tactical characteristics of track and field teaching. The basic mode of the cascade noise reduction self-encoder [26] is shown in Figure 5.

Through cascade noise reduction, the input of the self-encoder network is the result of noise distortion of the original data. The training process does not need any manually labeled data labels, and the target label is the pure original data itself. The criterion of network training is to minimize the difference between the output of the network and the pure original data, that is, the reconstruction loss. Suppose that *m* track teaching tactical characteristics quantify the training sample data set *x*={*x*^1^, *x*^2^,…*x*^*m*^} as network input, where *c* represents a pure original sample, while *x*^*n*^ represents the noise distortion result of the original sample *x*^*m*^. For a typical noise reduction autoencoder structure, the bottom-up process is usually called the encoding process, while the top-down process is known as the decoding process.

If *d* and *d*′ represent the weight matrix of the uplink and downlink encoder, and *b* and *b*′ represent the bias of the corresponding nodes, the objective function of the learned regularity term of a noise-reducing self-encoder is expressed as

(15) $$\begin{array}{c} \min\varepsilon =\eta {\sum_{i=1}^{m}\left\|x^{n}-x^{m}\right\|}_{2}^{2}+\nu \left(d^{2}\right)+{\left\|d^{\prime }\right\|}^{2}, \end{array}$$

where *ε* represents the set of all parameters to be learned in the network; *ν* is a nonlinear excitation function, and *η* is the weight factor of the parametric regular term used to balance the reconstruction loss and the weight penalty.

According to the characteristic data of track and field teaching tactics after noise reduction self-encoder training, input it into the constructed depth neural network. When the number of samples is enough, these samples can really reflect the characteristics of track and field teaching tactics. The output results of the final track and field teaching tactics evaluation are as follows:

(16) $$\begin{array}{c} Y\left(x^{n}\right)=\sum_{i=1}^{n}r\delta_{i}\left(x^{n}-x^{m}\right). \end{array}$$

Among them, *Y*(*x*^*n*^) represents the analysis result value, and *δ*_*i*_ represents the deviation coefficient of the output characteristics of teaching tactics.

## 4. Experimental Analysis

### 4.1. Experimental Scheme Design

In order to verify the effectiveness of the proposed method, an experimental analysis is carried out. In the experiment, the 20-kilometer walking race held by a university was taken as the research object, and 12 male athletes were selected as the research object. These 12 contestants were contestants in different classes of the same grade, aged between 18 and 19 years, and their physical qualities were relatively similar. In the 20-kilometer walking race, the characteristics of the speed change in the first and second half of the race are analyzed, and the feasibility of the analysis method in this paper is analyzed. Before the game, under the guidance of the coach, the training has been carried out for three months. The 12 people are divided into two groups. One group is the experimental group. Six players in this group compete in the tactics formulated by the coach, and the other group compete in accordance with their own training methods. Investigate the importance of the first and second half of the 20 km race walking race, as shown in Table 2.

According to the experimental scheme designed above, the effective analysis of the characteristics of teaching tactics is carried out. In the experiment, taking the analysis method of this paper, the method of [8], and the method of [9] for the 20 km race walking competition as the research object, analyze the error of different methods on the tactical analysis and the key degree of the influencing factors determined by the tactical characteristics as the experimental object, summarize the experimental data, and complete the experimental analysis.

### 4.2. Analysis of Experimental Results

In the experiment, firstly, the proposed method is analyzed. In the 20-kilometer race walking competition, the group using coach teaching tactics and the group without teaching tactics are used to determine the results of the 12 athletes. Among them, No. 1–6 are the athletes without coach teaching tactics, and 7–12 are the athletes under coach tactics. The 12 athletes complete the speed analysis of the 20-kilometer race walking competition. The results are shown in Table 4.

By analyzing the experimental results in Table 4, it can be seen that the shortest time spent by athletes No. 1–6 in the 20 km race walking competition is about 30.25 min, that of athletes No. 7–12 in the 20 km race walking competition is about 29.54 min, that of athletes No. 1–6 in the 20 km race walking competition is more than 30 min, and for athletes No. 7–12 in the 20 km race walking competition, two athletes spend less than 30 min, and the overall time is lower than that of athletes No. 1–6. It can be seen that the athletes using teacher tactics achieve better results, which verifies the feasibility of the proposed method.

**Figure 6:**
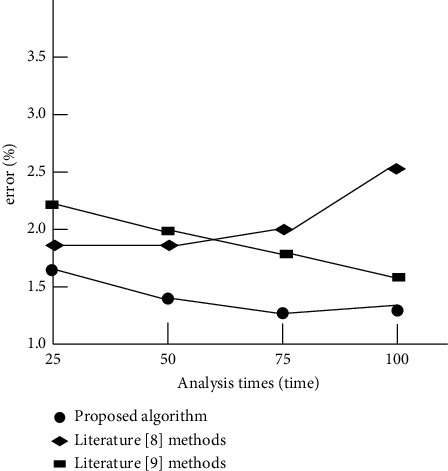
Comparison of analysis error results of different analysis methods.

The experiment further analyzes the error of the analysis method of this paper, the method of [8], and the method of [9] on the characteristics of teaching tactics in this race walking competition. The results are shown in Figure 6.

By analyzing the experimental results in Figure 6, it can be seen that there are some differences in the error of analyzing the characteristics of teaching tactics in this race walking competition by using the analysis methods of this paper, of [8], and of [9]. Among them, the analysis error of this method is low and is always lower than 1.8%, while the error of the two methods is high, which verifies the feasibility of the proposed method.

**Figure 7:**
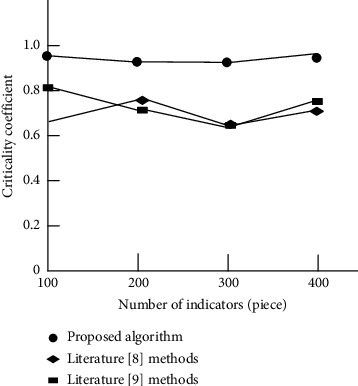
Analysis of key degree results of influencing factors determined by teaching tactics characteristics of different analysis methods.

The experiment further analyzes the key degree of the analysis method of this paper, the method of [8], and the method of [9] on the influencing factors determined by the characteristics of teaching tactics in this race walking competition. The results are shown in Figure 7.

By analyzing the experimental results in Figure 7, it can be seen that the key degrees of the influencing factors determined by the analysis method of this paper, the method of [8], and the method of [9] in this race walking competition are different. With the change of the number of indicators, the curve trend of the three methods has changed to some extent. Among them, the key coefficient of the influencing factors determined by the teaching tactical characteristics of the proposed method is higher and always higher than 0.9. Compared with the other two methods, the key coefficient is lower, which verifies the feasibility of the proposed method.

## 5. Conclusion

This paper designs a method to analyze the tactical characteristics of college track and field sports training teaching based on deep neural network. By analyzing the current situation of track and field sports training teaching in colleges and universities, this paper determines the key characteristics of teaching tactics. By determining the factors of teaching environment, the core competitiveness of track and field teams, and the teaching ability of track and field coaches, these factors are determined as the key characteristics, the data basis is analyzed, and the unified data quantitative processing is carried out to determine the key factor indexes affecting the analysis of tactical characteristics. This paper introduces the deep neural network to construct the evaluation model of the tactical characteristics of college track and field sports training teaching and further modifies the characteristic analysis results with the help of cascade noise reduction self-encoder to complete the analysis of the tactical characteristics of college track and field sports training teaching. The experimental results show that the proposed method can effectively analyze the teaching tactical characteristics of track and field sports training in colleges and universities and improve the performance of the evaluation of the teaching tactical characteristics of track and field sports training.

## Figures and Tables

**Table 1:** Contents of coaching ability indicators of track and field coaches.

Teaching ability	Content
Personal experience	Age, educational background, sports level, years, post training, etc.
Training ability	Diagnostic ability, prediction ability, designated plan ability, recovery ability, stimulation means, etc.
Teaching ability	Action modeling, explanation ability, sports equipment ability, learning ability
Competition guidance ability	Specify the program ability, organization and arrangement ability, and adjustment ability
Organization and management ability	Daily training and management, conflict resolution, etc.
Scientific research and innovation ability	Postgame summary, reflection ability, scientific research ability, etc.

**Table 2:** Investigation on the importance of the first and second half of the 20 km race walking race.

	The first-half result is even more important	The result in the second half is even more important	The first- and second-half results are equally important
Trainer	3	1	0
Percentage	75%	25%	0

The average speed (M/s) of 12 participating men in 20 km race walking is set as shown in Table 3.

**Table 3:** Average speed of participating men in 20 km race walking (m/s).

Number	Segmental average speed (km)					
1	0–2	2–4	4–6	6–12	12–16	16–20
2	3.95	4.12	4.16	4.35	4.38	4.43
3	3.95	4.12	4.17	4.35	4.35	4.43
4	3.96	4.13	4.18	4.35	4.35	4.42
5	3.96	4.14	4.17	4.32	4.36	4.41
6	3.95	4.13	4.16	4.32	4.39	4.40
7	3.95	4.12	4.15	4.33	4.35	4.40
8	3.96	4.12	4.16	4.34	4.32	4.43
9	3.94	4.13	4.16	4.33	4.36	4.39
10	3.94	4.15	4.17	4.34	4.36	4.35
11	3.95	4.16	4.18	4.35	4.38	4.32
12	3.94	4.17	4.19	4.35	4.35	4.33

**Table 4:** Analysis of 20 km race walking results.

Number	Result (min)	Number	Result (min)
1	30.25	7	29.54
2	32.15	8	30.21
3	33.21	9	29.65
4	33.22	10	30.12
5	31.25	11	30.14
6	30.45	12	30.54

## Data Availability

The raw data supporting the conclusions of this article will be made available by the author, without undue reservation.

## References

[1] Kasai N., Tanji F., Ishibashi A. (2021).

[2] Pollock N., Kelly S., Lee J., Stone B. (2021). A 4-year study of hamstring injury outcomes in elite track and field using the British Athletics rehabilitation approach. *British Journal of Sports Medicine*.

[3] Bing Z., Meschede C. (2020). Indirect and direct training of spiking neural networks for end-to-end control of a lane-keeping vehicle. *Neural Networks*.

[4] Zihao F. (2022). Complementing of sports training and PHYSICAL education. *Science and Technology Information*.

[5] Xinmiao N. (2021). ZHANG xin ’an, CAO lihua. Application of motion capture technology in sports field. *Sports Science and Technology*.

[6] Jinghua L. (2021). Design of sports training support System based on VR Technology. *Automation Technology and Application*.

[7] Adams W. M., Hosokawa Y., Casa D. J. (2021). Preseason heat safety in secondary school athletics. *Journal of Athletic Training*.

[8] Mengnan W. (2020). Chen kanggui. Application of inertial motion capture technology in sports training: a case study of badminton teaching. *Contemporary Sports Technology*.

[9] jiaqin X. (2020). *Application of simulation training method in track and field training [J] Sporting goods and technology*.

[10] Ling Q. (2021). Analysis on Influencing Factors of physical fitness training in track and field sprint. *Sports fashion*.

[11] Longo S., Cè E., Bisconti A. V. (2021). The effects of 12 weeks of static stretch training on the functional, mechanical, and architectural characteristics of the triceps surae muscle–tendon complex. *European Journal of Applied Physiology*.

[12] Marshall A. N., Mcleod T., Lam K. C. (2020). Characteristics of injuries occurring during cross-country: a report from the athletic training practice-based research network. *Journal of Athletic Training*.

[13] Drake J. A., Diggs L. P., Martin S. P. (2020). Characteristics of matriculants to thoracic surgery residency training programs. *The Annals of Thoracic Surgery*.

[14] Zhao J., Wang Y., Zhao D., Lizhen Z., Peijie C., Xin X. (2020). Integration of metabolomics and proteomics to reveal the metabolic characteristics of high-intensity interval training. *Analyst*.

[15] Ali R., Wharton R., Li L., Waterman J. (2021). 932Delivering excellence in orthopaedic training - a five year qualitative study of characteristics valued by trainees voting for trainer of the year. *British Journal of Surgery*.

[16] Lévesque J., Rivaz H., Rizk A., Frenette S., Boily M., Fortin M. (2020). Lumbar Multifidus Muscle Characteristics, Body Composition, and Injury in University Rugby Players. *Journal of Athletic Training, Body Composition, and Injury in University Rugby Players*.

[17] Alya Ev S., Shahriari M., Pardo D. (2021). Modeling extra-deep EM logs using a deep neural network. *Geophysics*.

[18] Junya Z. (2022). Zhang qiang, dai Yue. An intelligent walking robot construction method based on physiological neural network [J]. *Chinese Journal of Medical Physics*.

[19] Haifang Mu, Kai G., Hu B., Fuzzy P. I. D., Network N. (2022). *Impedance Control for Upper Limb of Rehabilitation Robot*.

[20] Xiao L. (2019). Design of a machine learning framework for sports outcome prediction. *Automation technology & application*.

[21] Lee S., Kim I. (2021). DVC㎞et: a deep neural network model for dense video captioning. *IET Computer Vision*.

[22] Alhnaity B., Kollias S., Leontidis G., Shouyong J., Bert S., Simon P. (2021). An autoencoder wavelet based deep neural network with attention mechanism for multi-step prediction of plant growth. *Information Sciences*.

[23] Yang X., Ni W., Yan W. (2021). 3-D electromagnetic-model-based absolute attitude estimation using a deep neural network. *Remote Sensing Letters*.

[24] Lee Y., Fang Y. C., Tien C. H. (2021). Deep neural network for coded MaskCryptographical imaging. *Applied Optics*.

[25] Renjun Z., Wang w. (2021). Simulation of network traffic prediction model based on deep neural network. *Computer simulation*.

[26] Dibiase R. M., Salas R., Gamaldo C. E. (2021). Training in neurology: implementation and evaluation of an objective structured clinical examination tool for neurology post-graduate trainees in lusaka, Zambia. *Neurology*.

